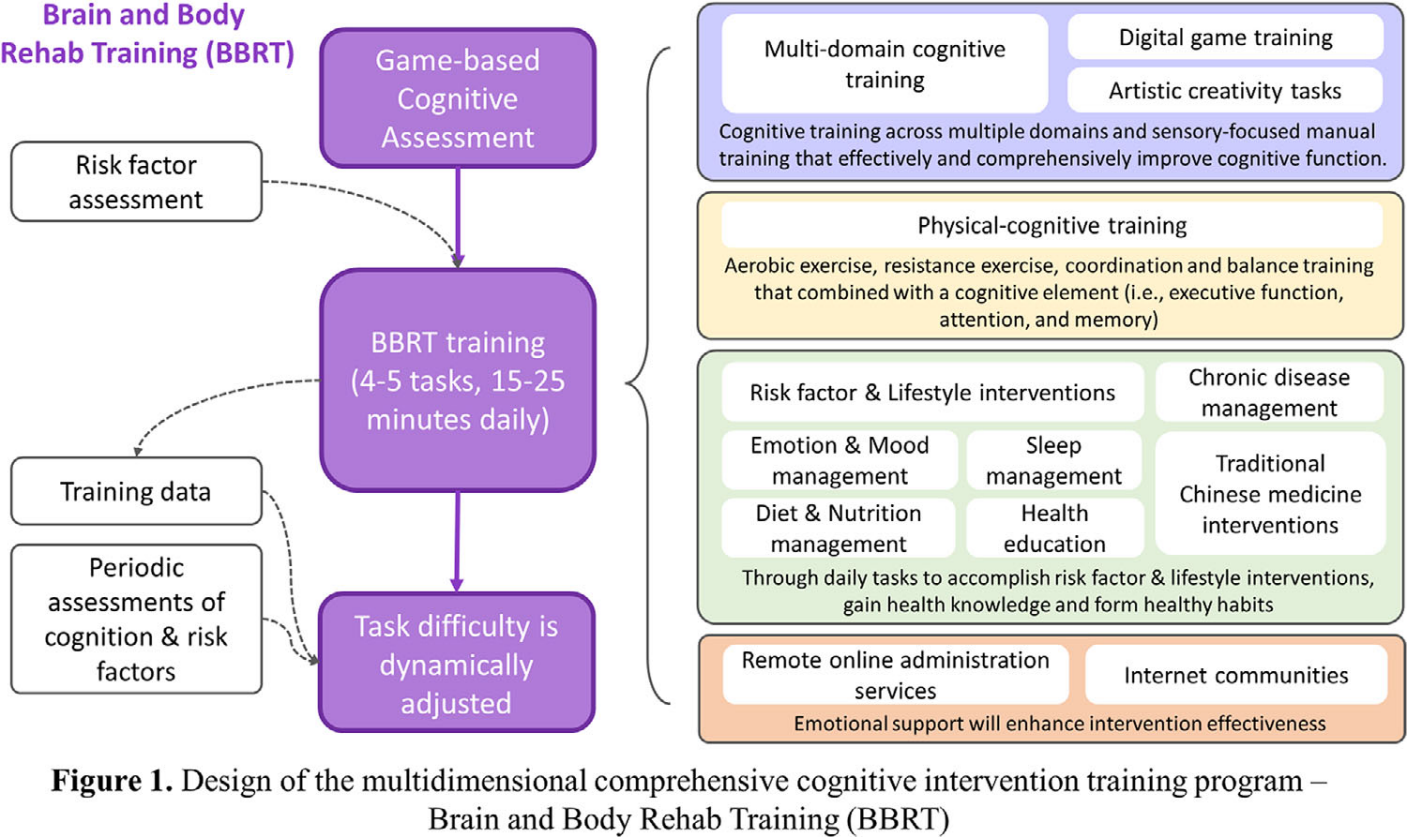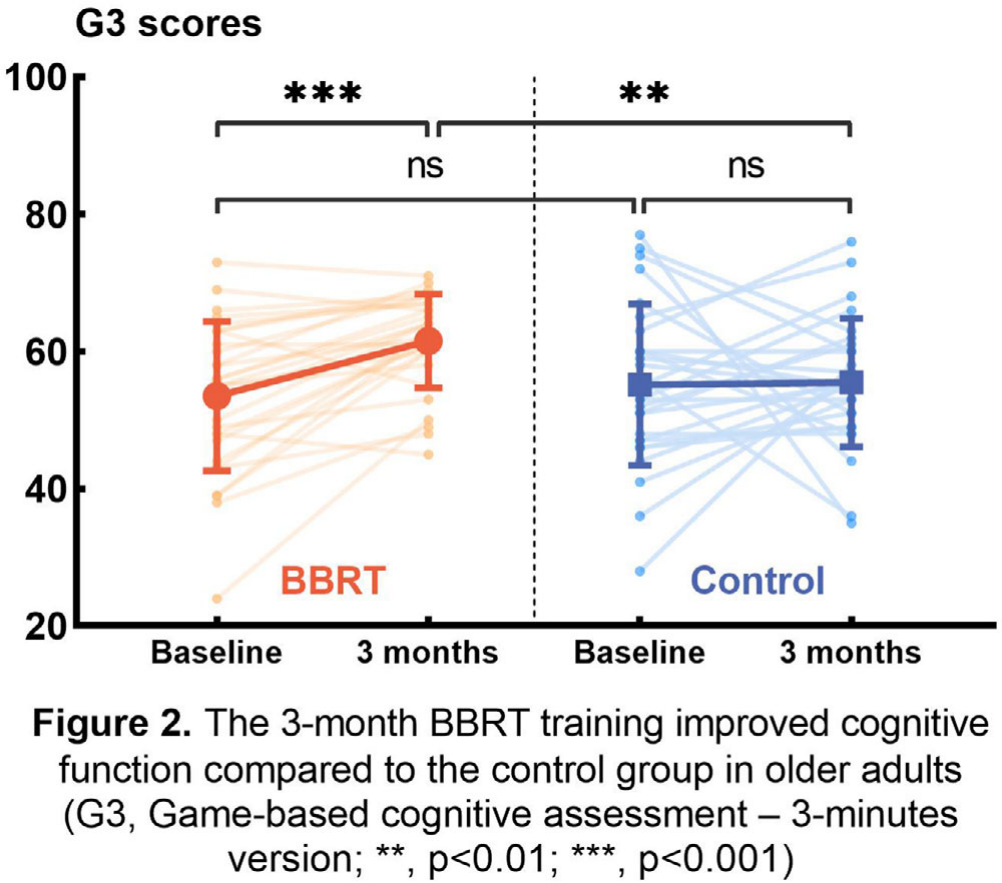# A Multidimensional Comprehensive Cognitive Intervention Training Program: Introduction of a Non‐Pharmacological Digital Therapeutic and Preliminary Results of Effectiveness on Cognitive Function

**DOI:** 10.1002/alz.094257

**Published:** 2025-01-09

**Authors:** Yatian Li, Jingnan Wu, Zhixing Zhou, Nan Chen, Huanhuan Xia

**Affiliations:** ^1^ Shanghai Bestcovered Limited, Shanghai China

## Abstract

**Background:**

Older adults with cognitive impairments will benefit from multicomponent interventions include cognitive training, exercise, and lifestyle modifications. However, many existing digital therapeutic products predominantly focus on computerized cognitive training, lacking effective approaches to other crucial interventions. We proposed a multidimensional comprehensive cognitive intervention training program – Brain and Body Rehab Training (BBRT), which integrates multidomain cognitive training with physical‐cognitive training and multidimensional lifestyle interventions and developed the digital therapeutic product – BBRT‐online based on WeChat mini‐program. The present study was to assess the effectiveness of BBRT in older adults with subjective memory impairments.

**Method:**

Using the WeChat mini‐program platform, we developed the BBRT‐online digital therapeutics product. Prior to the intervention, users undergo Game‐based Cognitive Assessment – Three‐Minute Version (G3). Subsequently, an individualized training program is assigned consisting of completing four to five daily tasks, including cognitive training, physical‐cognitive training, lifestyle interventions, chronic disease/diet/sleep/emotion management, and traditional Chinese medicine non‐pharmacological interventions among others (Figure 1). The intervention duration ranges 15‐25 minutes per day, and task difficulty is dynamically adjusted based on individual task performance and periodic cognitive assessments. Additionally, remote online administration services and internet communities are strongly recommended to offer emotional support and enhance intervention effectiveness. Sixty older adults reporting subjective memory complaints were recruited, with 30 assigned to receive BBRT‐online training and the remainder serving as the control group. Cognitive function was evaluated using the G3 at baseline and three months later. T‐tests were conducted to assess the impact of BBRT‐online on cognitive function.

**Result:**

At baseline, there was no significant difference in G3 scores between the BBRT group (53.5±10.87) and the control group (55.1±11.77, p = 0.583). Following three months of intervention, the BBRT group demonstrated a significantly higher G3 score (61.5±6.85) compared to baseline (p<0.001, Figure 2). Conversely, no such difference was observed in the control group (55.5 ± 9.34, p = 0.911).

**Conclusion:**

The BBRT digital therapeutics enabled cognitive assessment and individualized cognitive interventions and significantly improved cognitive function in older adults. Further studies are required to evaluate its effectiveness.